# Supplementary material for: Design of Oligonucleotide Carriers: Importance of Polyamine Chain Length
Source: Polymers (Basel). 2018 Nov 23;10(12):1297. doi: 10.3390/polym10121297 (PMC6401700; doi:10.3390/polym10121297)
Supplement: Supplementary file 1 [file polymers-10-01297-s001.pdf]

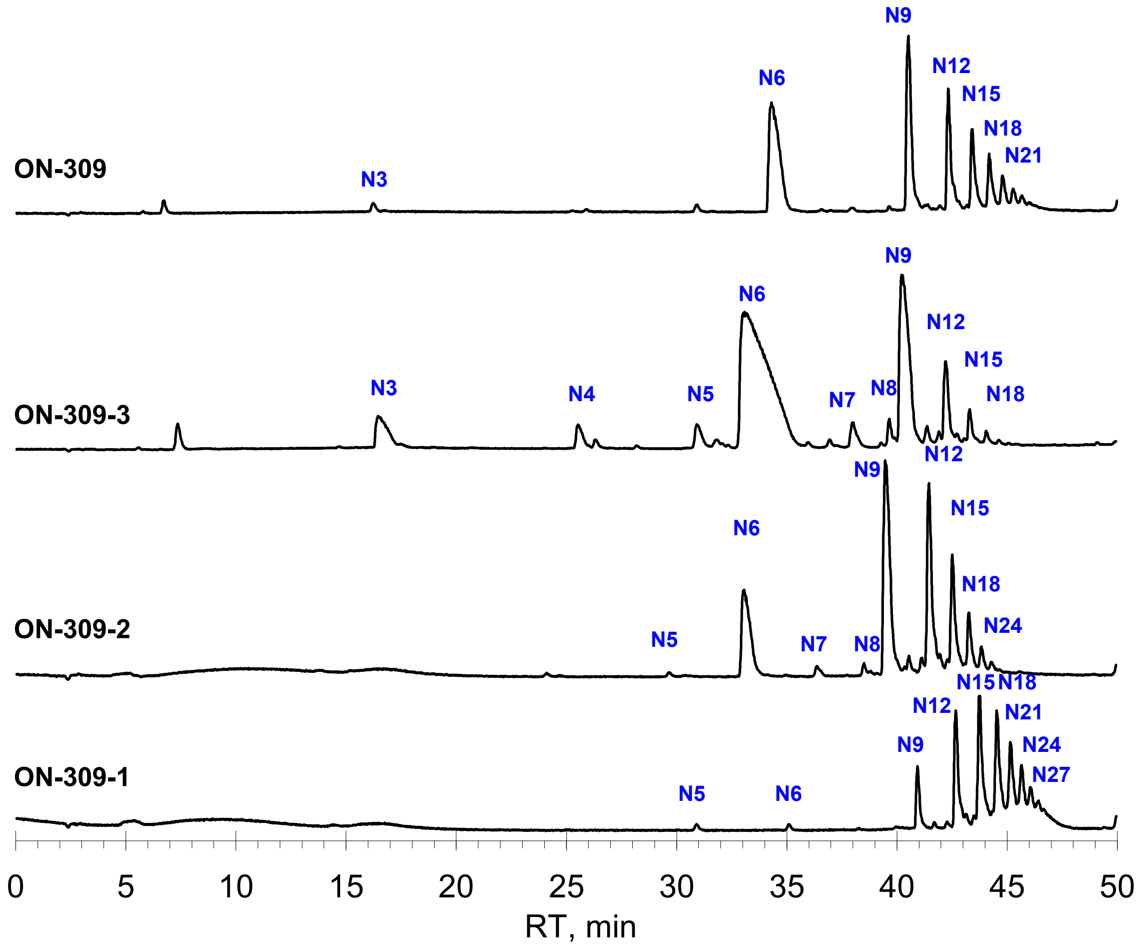

Figure S1. Liquid chromatography-mass spectrometry (LC-MS) analysis of ZS-309 fractions, isocratic elution with 0.1% solution of HFBA in water (A) and 0.1 % solution of HFBA in acetonitrile (B) (90% A / 10% B, sample concentration 50 mg·L^{-1}).

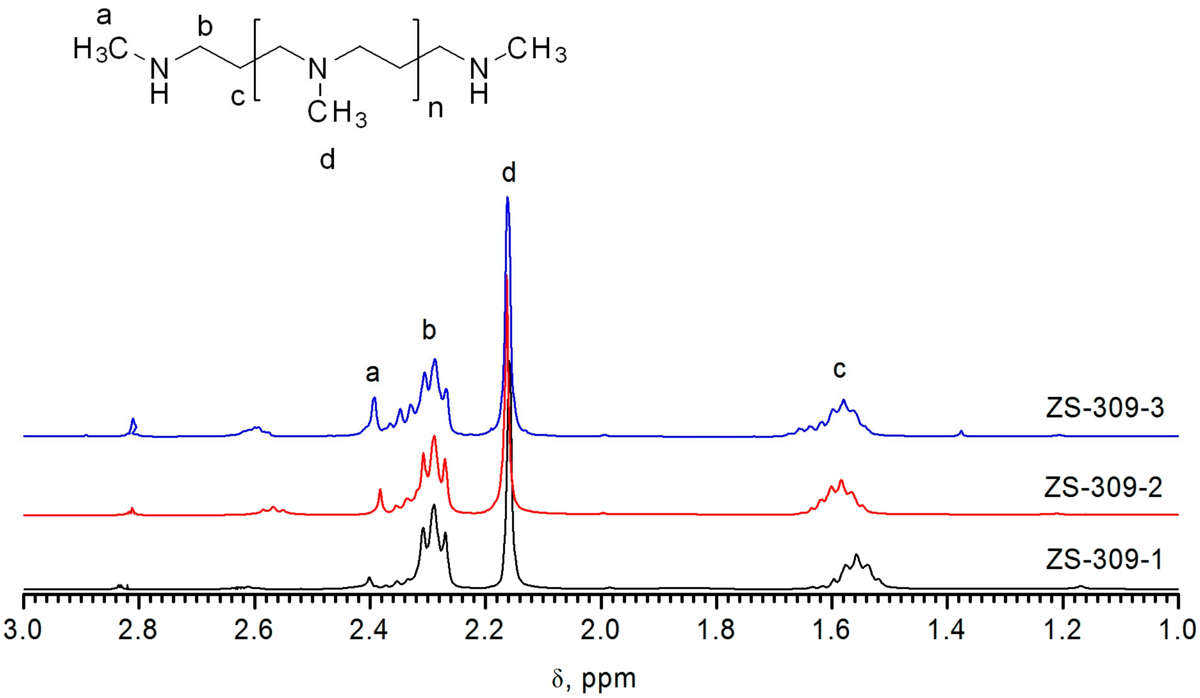

Figure S2. ^{1}H NMR spectra of LCPA (ZS-309) fractions.

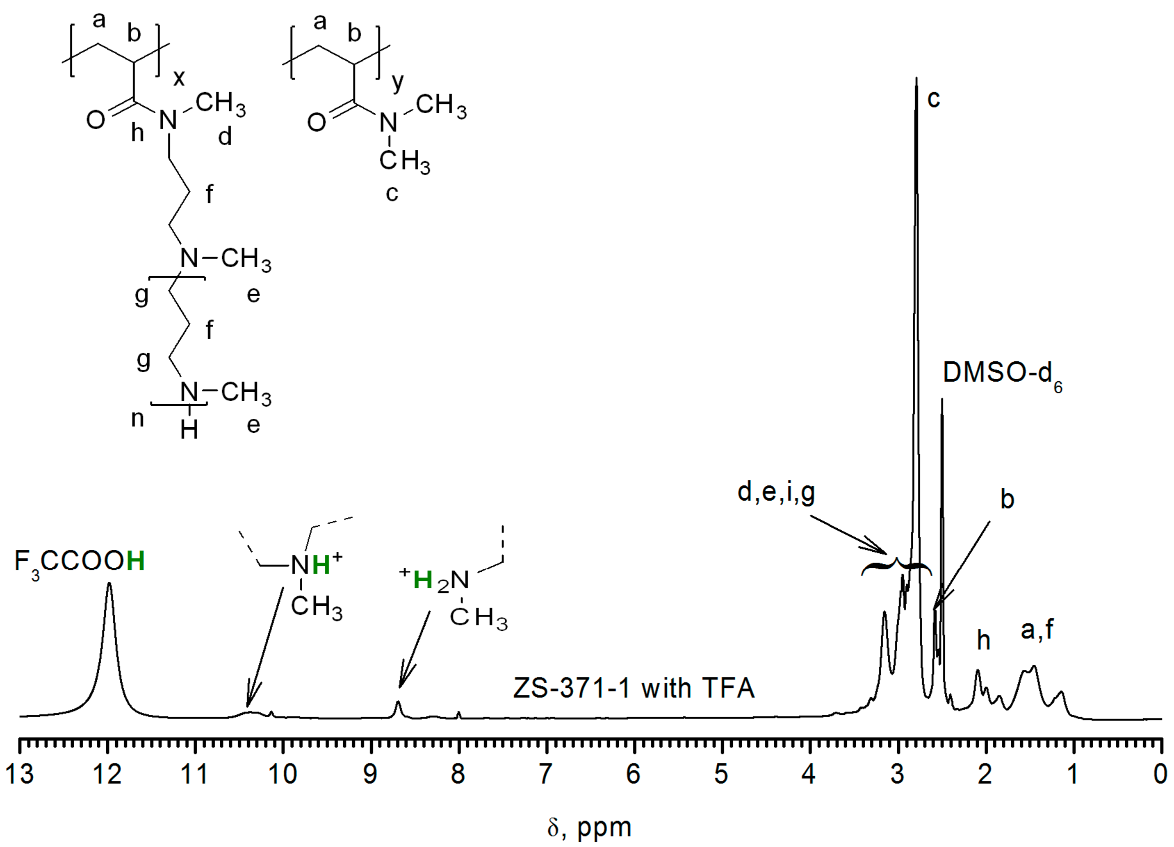
a
b
x
y
O
h
N
CH3
d
CH3
c
f
N−CH3
g
e
H
n
F3CCOOH
NH+
+H2N
d,e,i,g
DMSO-d6
ZS-371-1 with TFA
13
12
11
10
9
8
7
6
5
4
3
2
1
0
δ, ppm

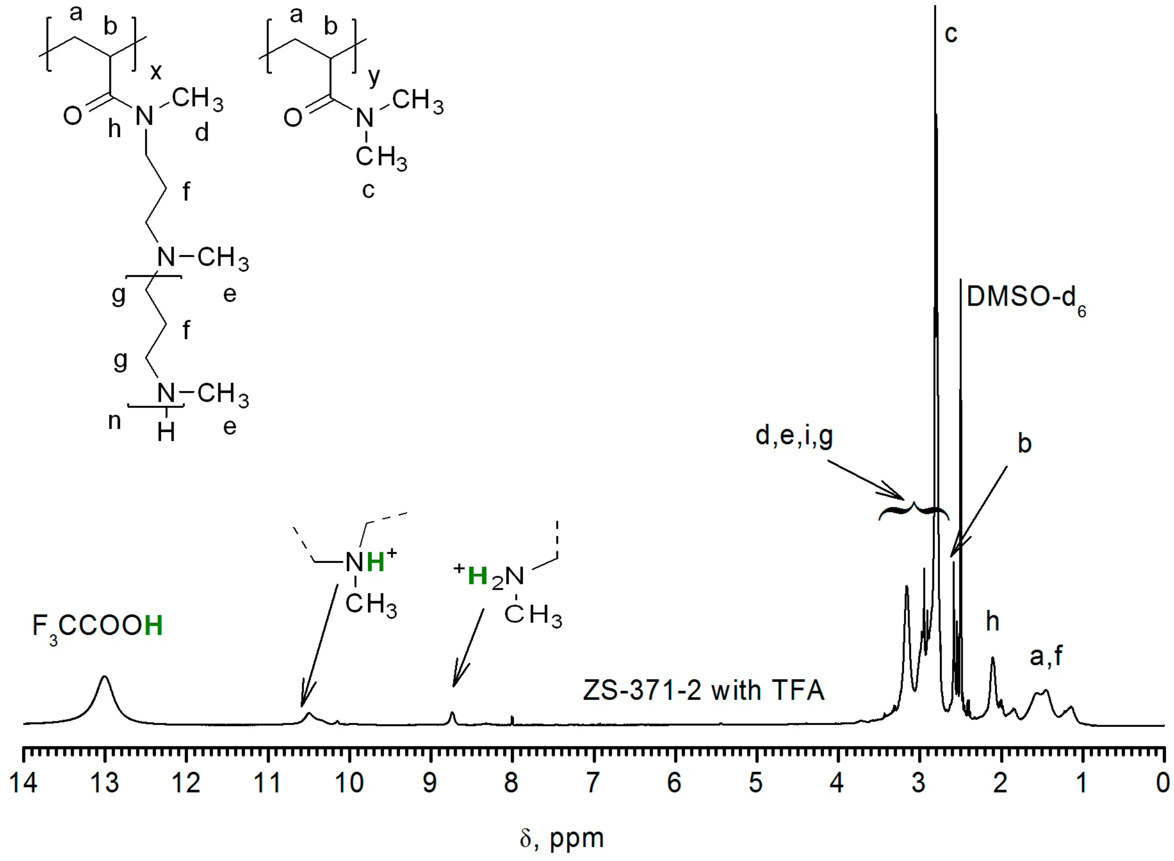
a
b
x
y
O
h
N
CH3
d
CH3
c
f
N−CH3
g
e
H
n
F3CCOOH
NH+
+H2N
d,e,i,g
DMSO-d6
ZS-371-2 with TFA
14
13
12
11
10
9
8
7
6
5
4
3
2
1
0
δ, ppm

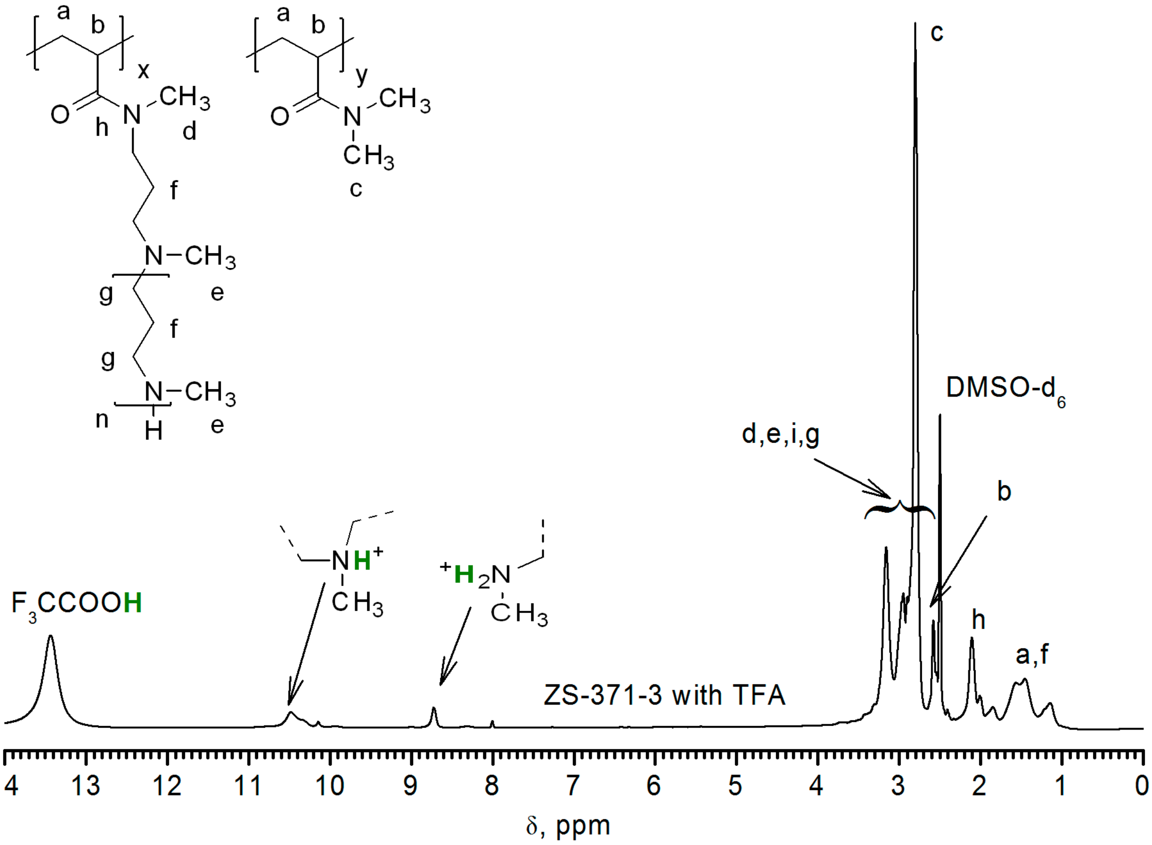

c
DMSO-d6
d,e,i,g
b
h
a,f
F3CCOOH
ZS-371-3 with TFA
δ, ppm

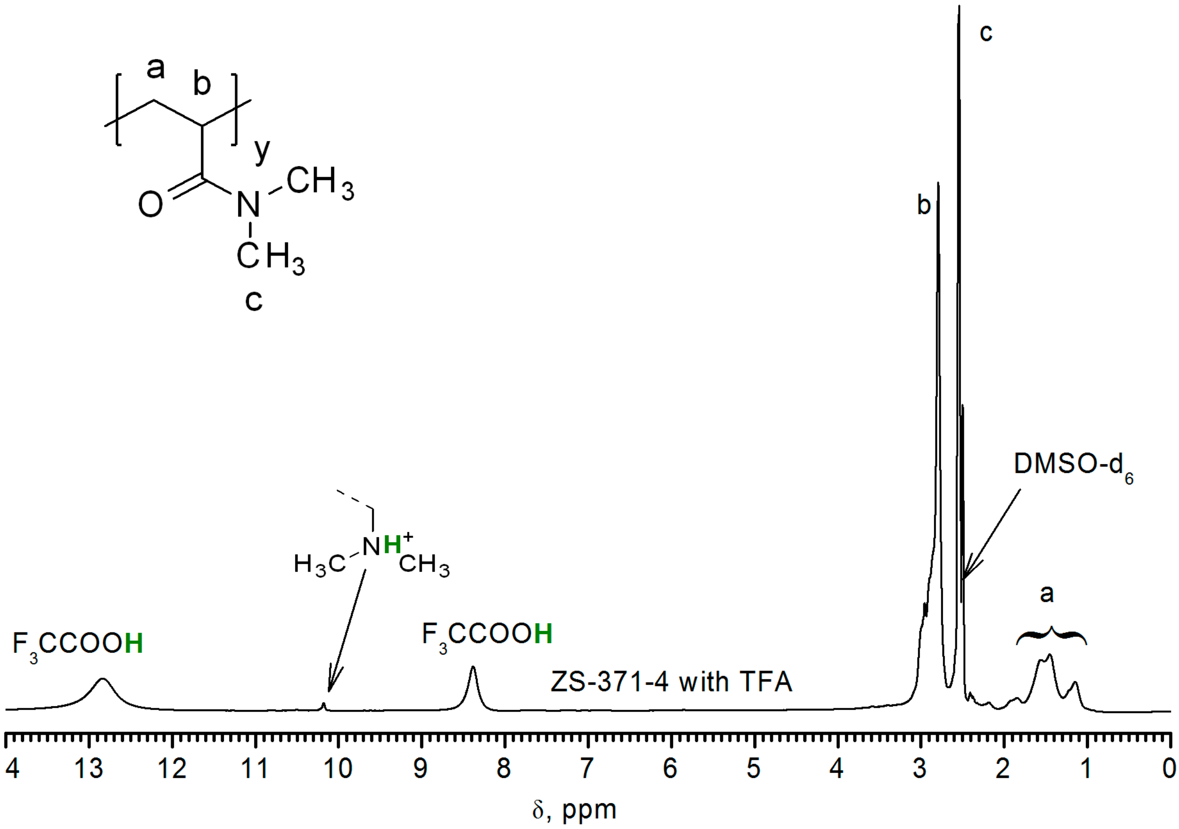

c
b
DMSO-d6
a
F3CCOOH
F3CCOOH
ZS-371-4 with TFA
δ, ppm

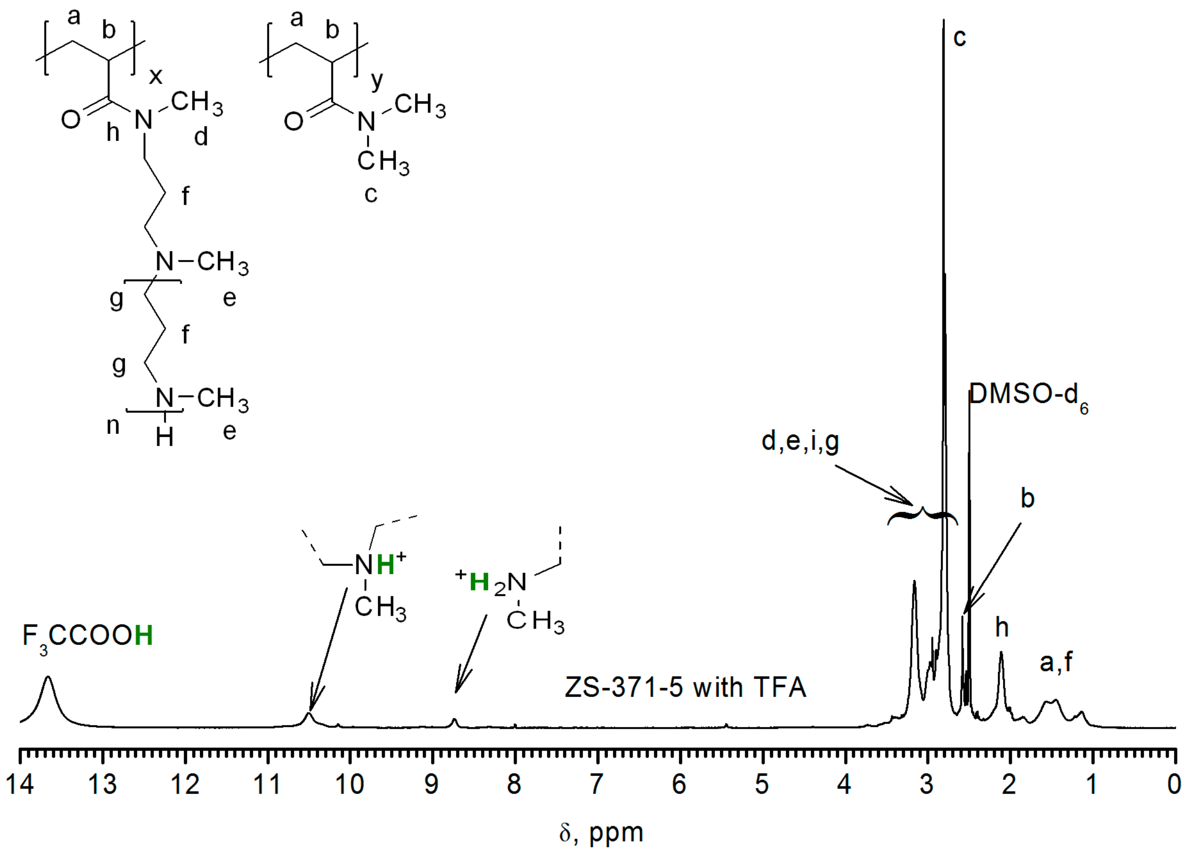

Figure S3. ^1H NMR spectra of copolymers (derivatized with TFA) in DMSO-d_6.
